# Thermal oxidation process on Si(113)-(3 × 2) investigated using high-temperature scanning tunneling microscopy

**DOI:** 10.3762/bjnano.13.12

**Published:** 2022-02-03

**Authors:** Hiroya Tanaka, Shinya Ohno, Kazushi Miki, Masatoshi Tanaka

**Affiliations:** 1 Graduate School of Engineering, Yokohama National University, 79-5 Tokiwadai, Hodogaya-ku, Yokohama 240-8501, Japan; 2 Department of Electrical Materials and Engineering, University of Hyogo, Shoya 2167, Himeji, Hyogo 671-2280, Japan; 3 National Institute for Materials Science, 1-1 Namiki, Tsukuba, Ibaraki 305-0044, Japan

**Keywords:** high-index Si surface, in situ measurement, oxidation, scanning tunneling microscopy (STM)

## Abstract

Thermal oxidation of Si(113) in a monolayer regime was investigated using high-temperature scanning tunneling microscopy (STM). Dynamic processes during thermal oxidation were examined in three oxidation modes – oxidation, etching, and transition modes – in the third of which both oxidation and etching occur. A precise temperature–pressure growth mode diagram was obtained via careful measurements for Si(113), and the results were compared with those for Si(111) in the present work and Si(001) in the literature. Initial oxidation processes were identified based on high-resolution STM images.

## Introduction

High-index silicon surfaces have drawn considerable interest for their usefulness in three-dimensional metal oxide semiconductor field-effect transistors (MOSFETs) [1]. Here, formation processes of ultrathin SiO_2_ at the interface are considered to be quite important in determining its dielectric properties. To study procedures to fabricate gate dielectrics, it will be necessary to understand thermal oxidation on silicon surfaces as well as metal-induced oxidation and silicidation [2]. Nevertheless, dynamic processes in oxidation have been studied scarcely so far, especially for high-index silicon surfaces. The study of the oxides grown on high-index silicon surfaces is of great significance because the corners of the Fin-type FETs should have such surfaces, where the electric field is enhanced, which significantly affects the device performance [3].

Recently, the observation of oxidation at the atomic level in both real time and real space has been recognized as an important experimental challenge toward elucidating the dynamic processes in oxidation. For example, the formation processes of iron oxide nanoparticles have been studied in detail using state-of-the-art X-ray scattering methods [4]. As a complementary method, variable-temperature scanning tunneling microscopy (VT-STM) has been used as the ideal tool to investigate reaction dynamics on surfaces. In our previous studies, we applied our VT-STM to investigate hydrogen diffusion [5] and titanium silicide formation [6] on Si(001).

In our previous studies of oxidation on Si(113), we investigated the electronic states of the Si 2p and O 1s core levels in detail during thermal oxidation using high-resolution X-ray photoelectron spectroscopy with synchrotron radiation [7–9]. Using a state-of-the-art wet oxidation procedure, we also reduced the interface trap density (*D*_it_) at the SiO_2_/Si interface on the Si(113) substrate dramatically, so that it was close to that of Si(001) [1]. To gain insight into the origin of this remarkable reduction in *D*_it_, it is necessary to understand the dynamic processes that occur during thermal oxidation more precisely. To this end, we observed the reaction dynamics during thermal oxidation on Si(113) using our VT-STM.

## Experimental

The Si(113) sample used was a p-type (B-doped) wafer with a resistivity of 1–10 Ω·cm. Sacrificed oxidation was performed to produce the oxide with a thickness of 200 nm using an oxidation furnace. The sample was cut to 6.5 × 1.5 mm^2^, rinsed with acetone to remove the oily materials, and rinsed with a mixture of sulfuric acid and hydrogen peroxide to remove the organic materials. It was then etched with a mixture of hydrochloride and hydrogen peroxide to form an ultrathin oxide layer before it was introduced to an ultrahigh-vacuum (UHV) chamber. The sample was degassed over 12 h at 870 K and then cleaned by flashing to 1370 K for 10 s several times.

The sample was observed at both room temperature (RT) and high temperatures. To reduce contamination effectively, we used a tip made of PtIr or Nb for the measurement at RT and a tip made of W for the measurements at high temperatures. The PtIr and Nb tips were rinsed with acetone and simply cut using a nipper. The W tip was also cut using a nipper before it was introduced to the UHV chamber. It was then heated to 1270 K with electron bombardment to remove the oxide.

Experiments were carried out in a UHV chamber with an STM apparatus (JEOL, JSTM-4500XT). The base pressure of the chamber equipped with the STM unit was kept at 5.0 × 10^−9^ Pa. For the high-temperature measurements, in order to make the temperature of the tip close to that of the sample, the STM tip was moved close (100 μm) to the sample and was kept in that position for a while before the measurements were started. Pure oxygen gas was introduced using a variable leak valve. It took about 3–30 s to obtain each STM image so that the amount of oxygen exposure would differ slightly between the upper and lower parts of the STM image. We put the average value of oxygen exposure at the corner of the STM images when necessary.

## Results and Discussion

### Clean surface

**Figure 1:**
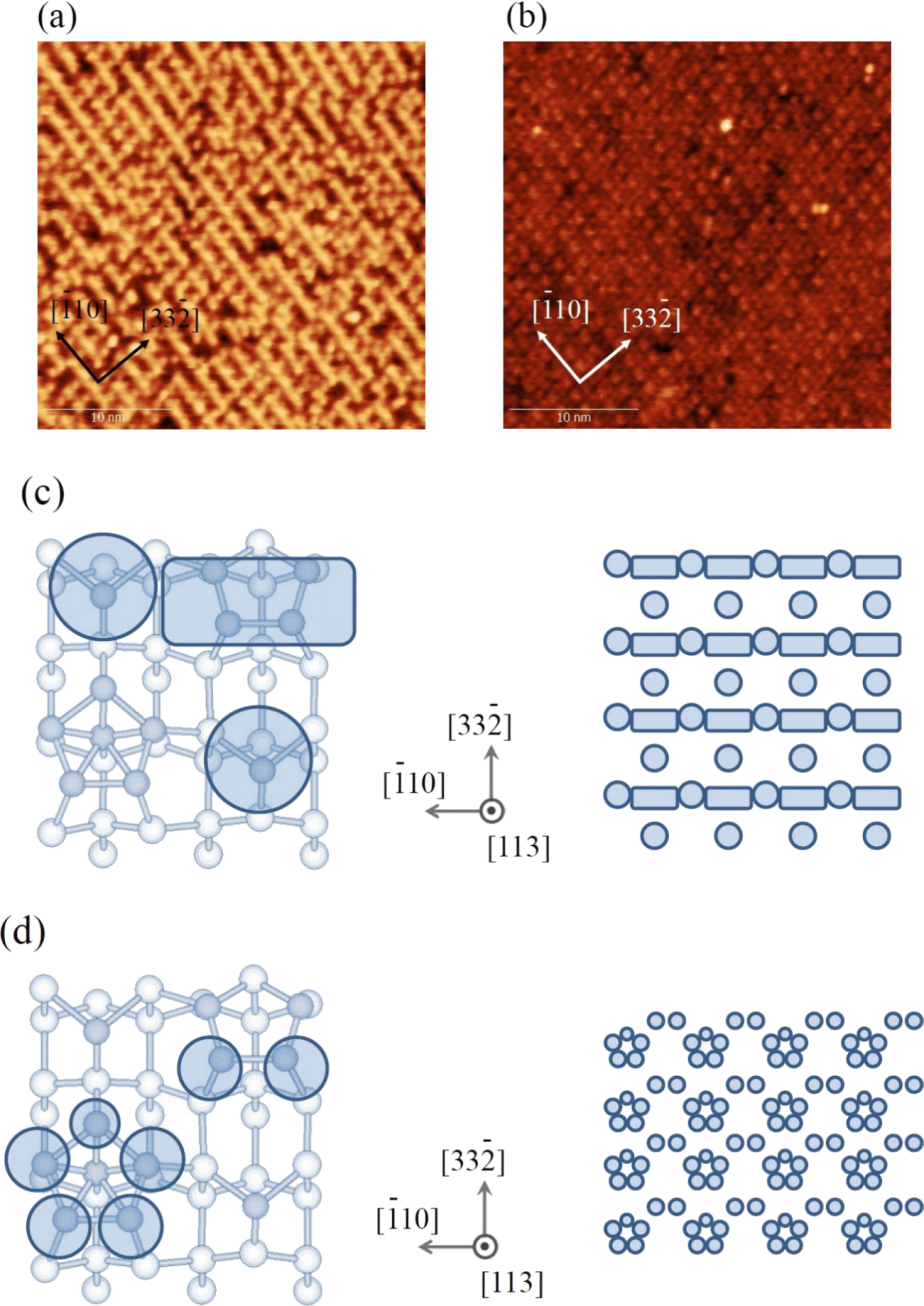
(a) Filled-state STM image of Si(113)-(3 × 2). Image size: 30 × 30 nm^2^, *V*_s_ = −2.0 V, *I*_t_ = 0.05 nA. (b) Empty-state STM image of Si(113)-(3 × 2). Image size: 30 × 30 nm^2^, *V*_s_ = +1.5 V, *I*_t_ = 0.05 nA. (c) Shaded area superimposed on the schematic of Si(113)-(3 × 2) represents the bright area observed in the filled-state STM image. The periodic structure formed by these bright areas is shown in the right panel. (d) Shaded area superimposed on the schematic of Si(113)-(3 × 2) represents the bright area observed in the empty-state STM image. The periodic structure formed by these bright areas is shown in the right panel.

Figure 1a,b show filled-state and empty-state STM images of a clean Si(113)-(3 × 2) surface observed at RT, respectively. The appearance of the STM images was reasonably interpreted by Hara et al. [10] based on the structure model proposed by Dąbrowski and co-workers [11], as shown in Figure 1c,d, corresponding to the filled-state and empty-state STM images, respectively. In the Dąbrowski model, two types of pentamer exist: one incorporates a Si atom at the center (i-pentamer) and the other does not (n-pentamer), as denoted by [12]. In the filled-state image, there are long lines along the 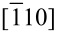 direction, which correspond to the n-pentamer site and the adatom site. In between the long lines there are the other adatom sites, which appear as isolated protrusions. In the empty-state image, the i-pentamer site is predominantly highlighted by the additional protrusions at the edge of the n-pentamer site. Our present results are consistent with the STM data in the previous works [10,12], as well as with those observed at high temperatures [13].

### Oxidation regime

**Figure 2:**
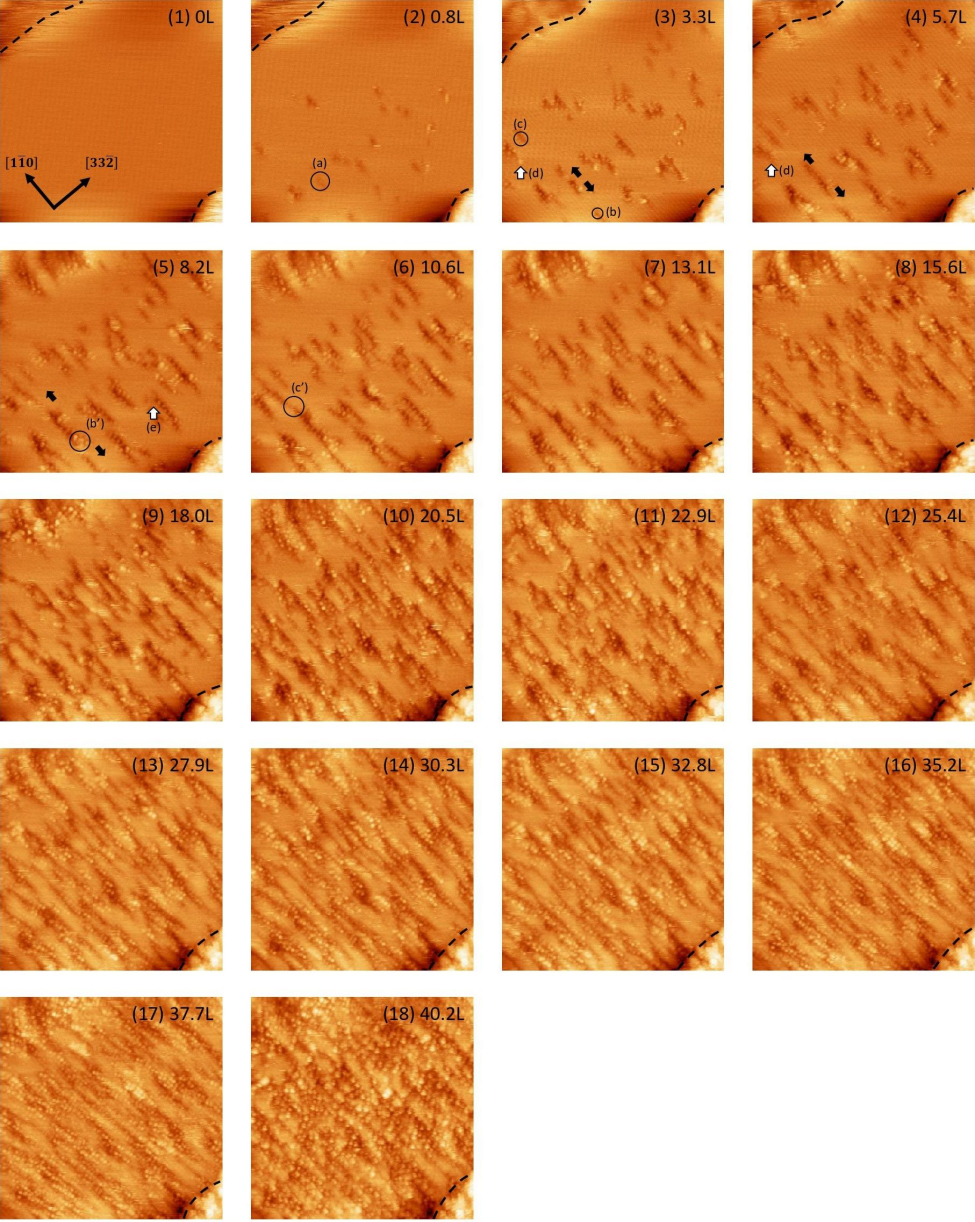
A series of filled-state STM images of Si(113)-(3 × 2) upon oxidation at an oxygen pressure of 1.3 × 10^−5^ Pa and a sample temperature of 820 K. A Langmuir unit is defined such that 1.0 L = 1.33 × 10^−4^ Pa·s. Image size: 40 × 40 nm^2^, *V*_s_ = −1.6 V, *I*_t_ = 0.07 nA.

Figure 2 shows a series of STM images of the filled state for oxidation at an oxygen pressure of 1.3 × 10^−5^ Pa and a sample temperature of 820 K. Under these conditions, only oxidation occurs; etching does not. The circle (a) shows one of the initial oxidation sites, appearing as a dark depression on the terrace. It is noted that the nucleation sites of the oxide appear randomly scattered at the middle part of the terrace. There are steps initially both at the upper-left corner and lower-right corner, as indicated by the dotted lines. The circles (b) and (c) also show the initial oxidation sites. The oxide initially formed at site (a) is expanded preferentially along the 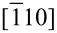 direction, as indicated by the solid arrows, and merges with the oxides initially formed at sites (b) and (c), as shown by circles (b’) and (c’). The empty arrow (d) shows a bright protrusion formed at the edge of the oxide. The empty arrow (e) shows protrusions formed inside the oxide.

In the case of Si(001), it was reported that a silicon atom can be removed from the surface during thermal oxidation and expelled onto the surface [14]. The protrusion seen at site (d) may be such expelled silicon atoms. In contrast, protrusions seen inside the oxide should correspond to the silicon atoms of the Si(113)-(3 × 2), as shown in Figure 1. We will discuss the details of these protrusions later based on magnified STM images, obtained under similar oxidation conditions.

From the present results, preference for oxidation is not clear based on the position of the steps. However, it should be noted that the step position indicated by the broken line at the upper-left corner changes significantly, indicating the decoration by the silicon atoms. Such a process is plausible because the silicon atoms on the substrate can be expelled via the oxidation process and can migrate on the terrace, as suggested for Si(001) [14]. Although such mobile silicon atoms cannot be visualized directly, some of the aforementioned protrusions also indicate the expelled silicon atoms. The step position becomes unclear above the oxygen exposure of ≈8 L. Afterwards, the oxidation may occur at the extended terrace formed by the decorated silicon atoms. In contrast, the step position indicated by the broken line remains almost constant at the lower-right corner. Later, we will report that the step position can be pinned by the oxide.

### Oxidation and etching regime

**Figure 3:**
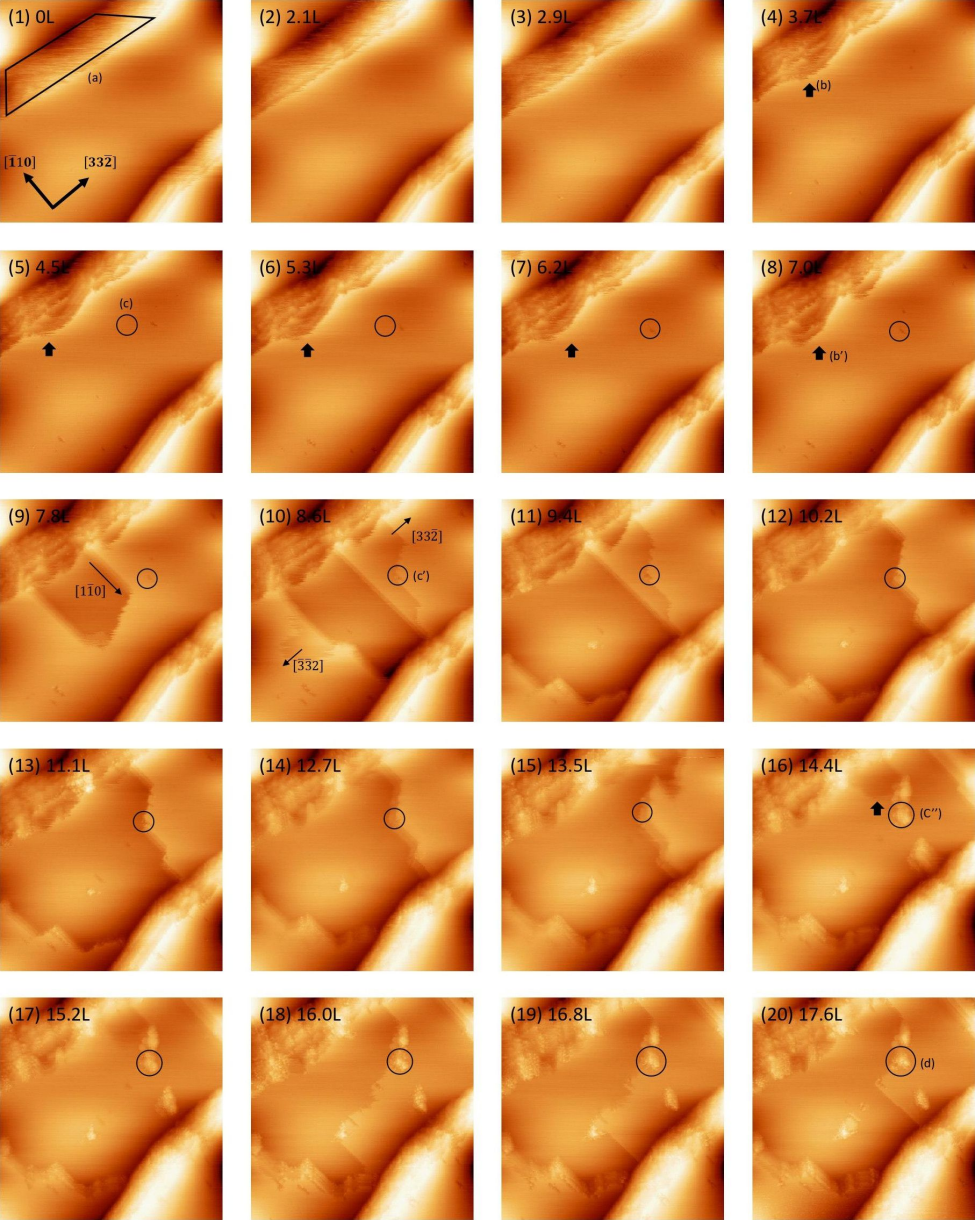
A series of filled-state STM images of Si(113)-(3 × 2) upon oxidation at an oxygen pressure of 1.3 × 10^−6^ Pa and a sample temperature of 820 K. A Langmuir unit is defined such that 1.0 L = 1.33 × 10^−4^ Pa·s. Image size: 100 × 100 nm^2^, *V*_s_ = −2.0 V, *I*_t_ = 0.07 nA.

**Figure 4:**
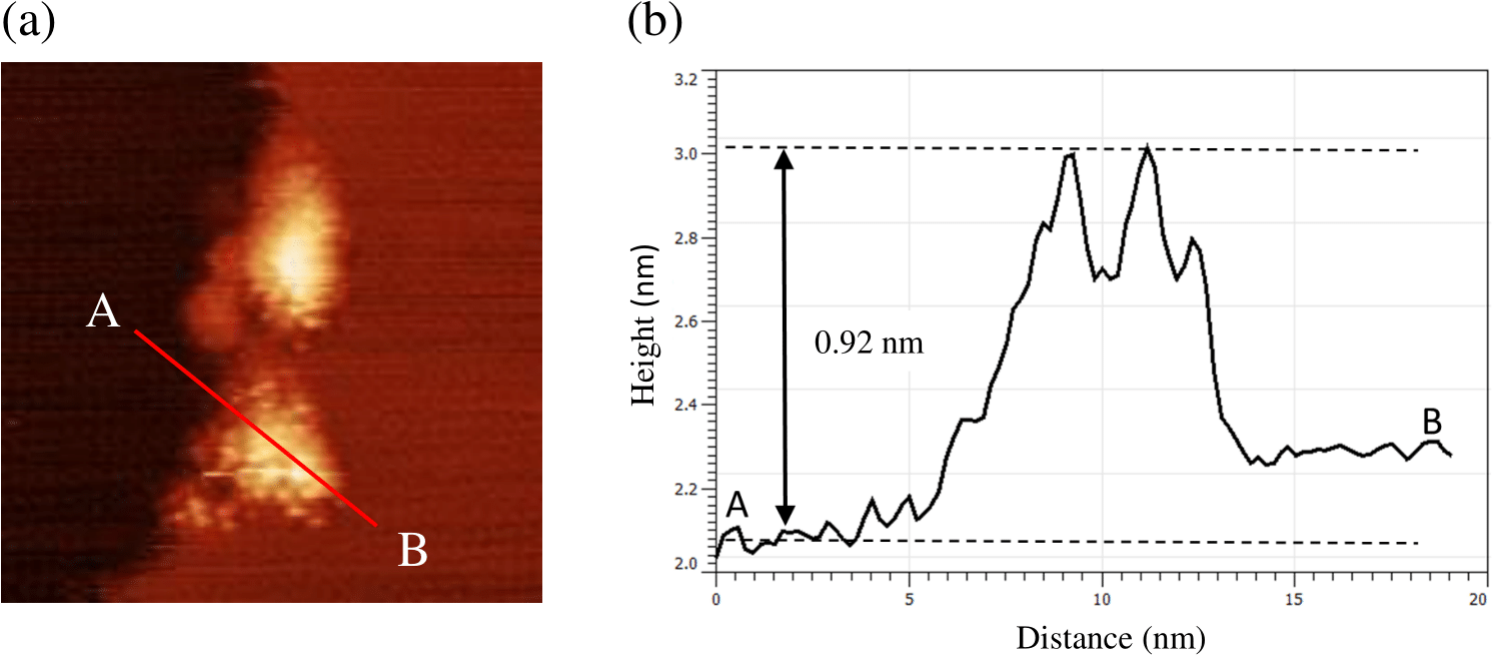
(a) Magnified image of the island in (d) of Figure 3. (b) Line profile along the line A–B in (a).

Figure 3 shows a series of STM images of the filled state for oxidation and etching at an oxygen pressure of 1.3 × 10^−6^ Pa and a sample temperature of 820 K. At the initial stage, the facet part in (a) is gradually removed, especially near the step edge, as indicated by the solid arrow (b). At the same time, a dark depression forms inside circle (c). This dark depression is similar to that observed in the oxidation mode. At an oxygen exposure of 7.0 L, the step-flow etching of the three-layer depth occurs along the 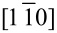 direction from the step edge indicated by the solid arrow (b’). At an oxygen exposure over 8.6 L, step-flow etching occurs along the 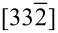 direction and in the opposite direction. The step-flow etching is pinned at the dark depression site corresponding to the oxide, as indicated by the circle (c’). After the step-etching proceeds, a small island remains, as shown in the circle (c”). The step-flow etching of the monolayer depth occurs just beside the remaining island, as shown by the solid arrow. The island remains at the step edge, as shown in the circle (d). A magnified image of the island in (d) is shown in Figure 4a. The line profile along the cross section A–B is shown in Figure 4b. The height corresponds to the fourth layer as measured from the lower terrace, corresponding to the occurrence of the consecutive step-flow etching of the three-layer depth and of the single-layer depth.

### Etching regime

**Figure 5:**
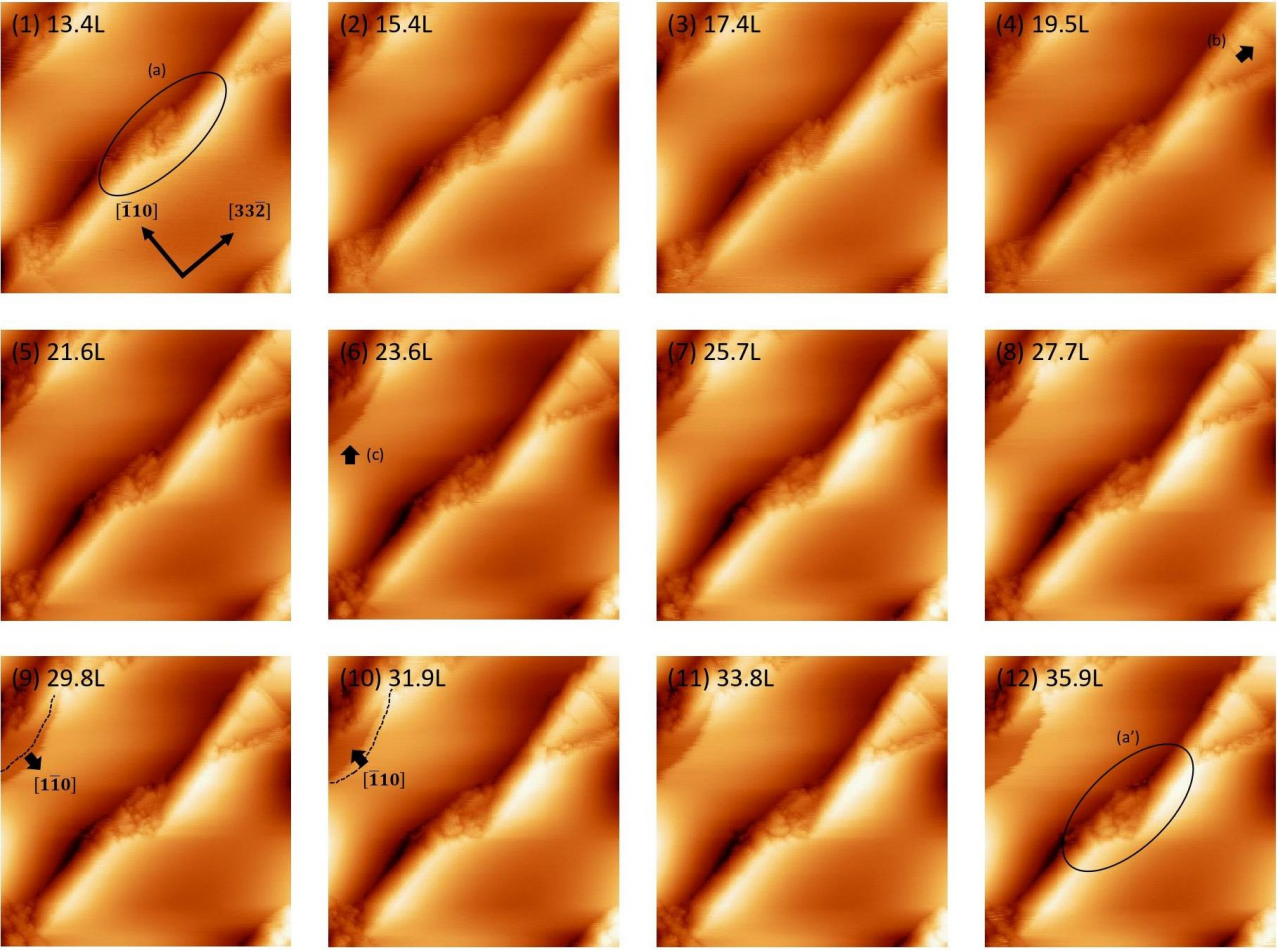
A series of filled-state STM images of Si(113)-(3 × 2) upon oxidation at an oxygen pressure of 6.5 × 10^−7^ Pa and a sample temperature of 820 K. A Langmuir unit is defined such that 1.0 L = 1.33 × 10^−4^ Pa·s. Image size: 100 × 100 nm^2^, *V*_s_ = −2.0 V, *I*_t_ = 0.07 nA.

Figure 5 shows a series of STM images of the filled state for oxidation and etching at an oxygen pressure of 6.5 × 10^−7^ Pa and a sample temperature of 820 K. The etching process occurs preferentially at the facet region indicated by the ellipse (a), resulting in a diminished volume as shown by the ellipse (a’). At the same time, step-flow etching occurs at sites indicated by the solid arrows (b) and (c). There is a case where faceting and step-flow etching occur consecutively. At an oxygen exposure of 29.8 L, step-flow etching occurs in the 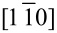 direction as indicated by the solid arrow. In contrast, the faceting or partial recovery of the step occurs in the opposite 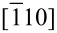 direction as indicated by the solid arrow, at an oxygen exposure of 31.9 L. It was reported that an attractive force is induced between the steps effectively on Si(113) at high temperatures [15]. Therefore, it is plausible that the fluctuation at the step edge is due to the competition between the step-flow etching and faceting associated with the attractive force between the steps. It is noted that the trend of step bunching with the steps along the 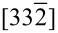 direction is consistent with the observation in the literature [16], which may be due to the effective attractive interaction between the steps [15]. No dark depression corresponding to the oxides was observed.

### Temperature–pressure growth mode diagram

**Figure 6:**
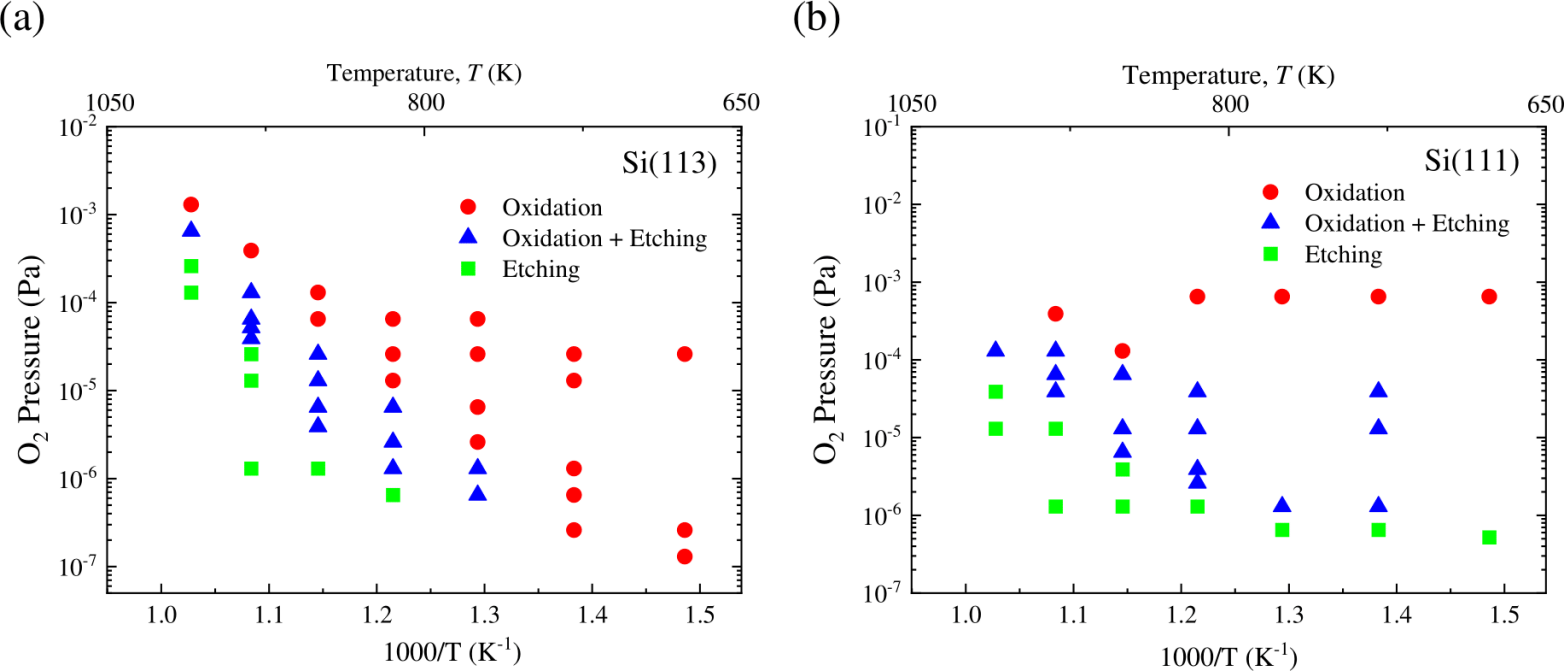
Temperature–pressure growth mode diagram for thermal oxidation on (a) Si(113) and (b) Si(111).

Figure 6a and Figure 6b show temperature–pressure growth mode diagrams of thermal oxidation on Si(113) and Si(111), respectively. Characterization of this region as a transition region in which both oxidation and etching occur is important because the surface roughness is enhanced in this mode [17]. It is noted that the oxidation and transition modes are classified as passive oxidation, and the etching mode is classified as active oxidation. It has been shown that the oxide-covered surface is smoother on Si(113) than on Si(001), especially in the transition region [17–18]. In the present work, we investigated the boundary lines between different growth modes precisely. It is clear that the transition region (oxidation and etching) is much narrower for Si(113) than for Si(001) [17] or Si(111).

It is considered that a mobile SiO species can migrate before desorption as metastable SiO or be incorporated into oxide islands [19]. Here, the residence time of a SiO species is postulated to be longer in order to produce larger oxide islands (two-dimensional islands as denoted in [19]). The present results suggest that the residence time of a mobile SiO species is relatively shorter on Si(113), leading to the less effective formation of oxide islands to enhance surface roughness. It is plausible that the narrow feature of the transition region for Si(113) is related to the observed flatness of the oxidized surface on Si(113) [18].

### Structure model of oxidized Si(113)-(3 × 2)

**Figure 7:**
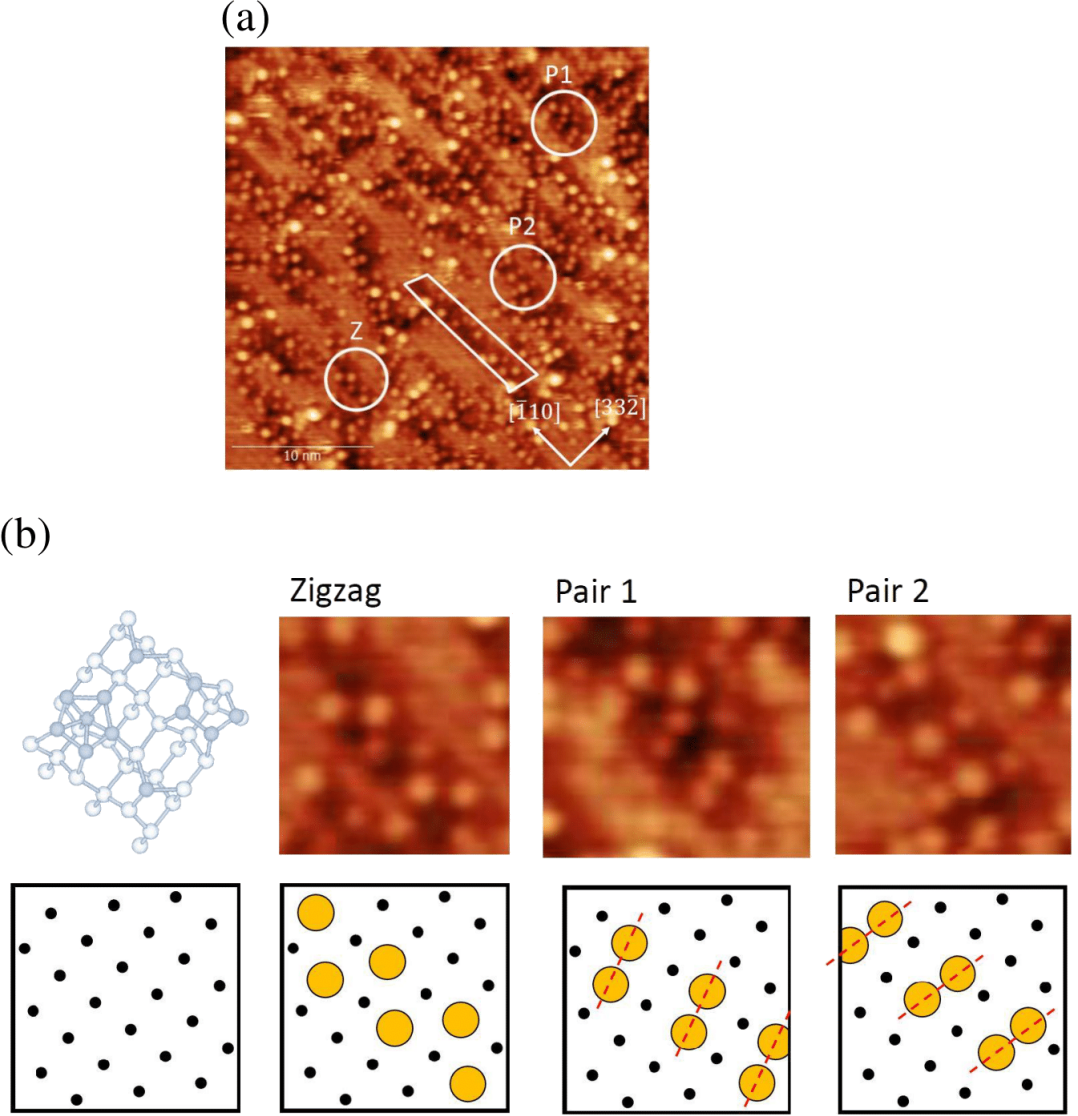
(a) High-resolution STM image of Si(113)-(3 × 2) upon oxidation at an oxygen pressure of 2.6 × 10^−5^ at 820 K, corresponding to the oxidation regime. Total oxygen exposure is 30 L. (b) Three areas showing typical patterns, denoted as Zigzag (Z), Pair 1 (P1), and Pair 2 (P2) in (a), are compared with the structure models.

A high-resolution STM image obtained for the oxidation regime is shown in Figure 7a. Here, the total oxygen exposure is 30 L. We found that the oxidized parts exhibit well-resolved bright protrusions. There are three patterns with reference to the (3 × 1) lattice: a zigzag pattern and two slightly tilted pairs, denoted here as Pair 1 and Pair 2, as shown in Figure 7b. It is noted that several tilted pairs tend to align together.

**Figure 8:**
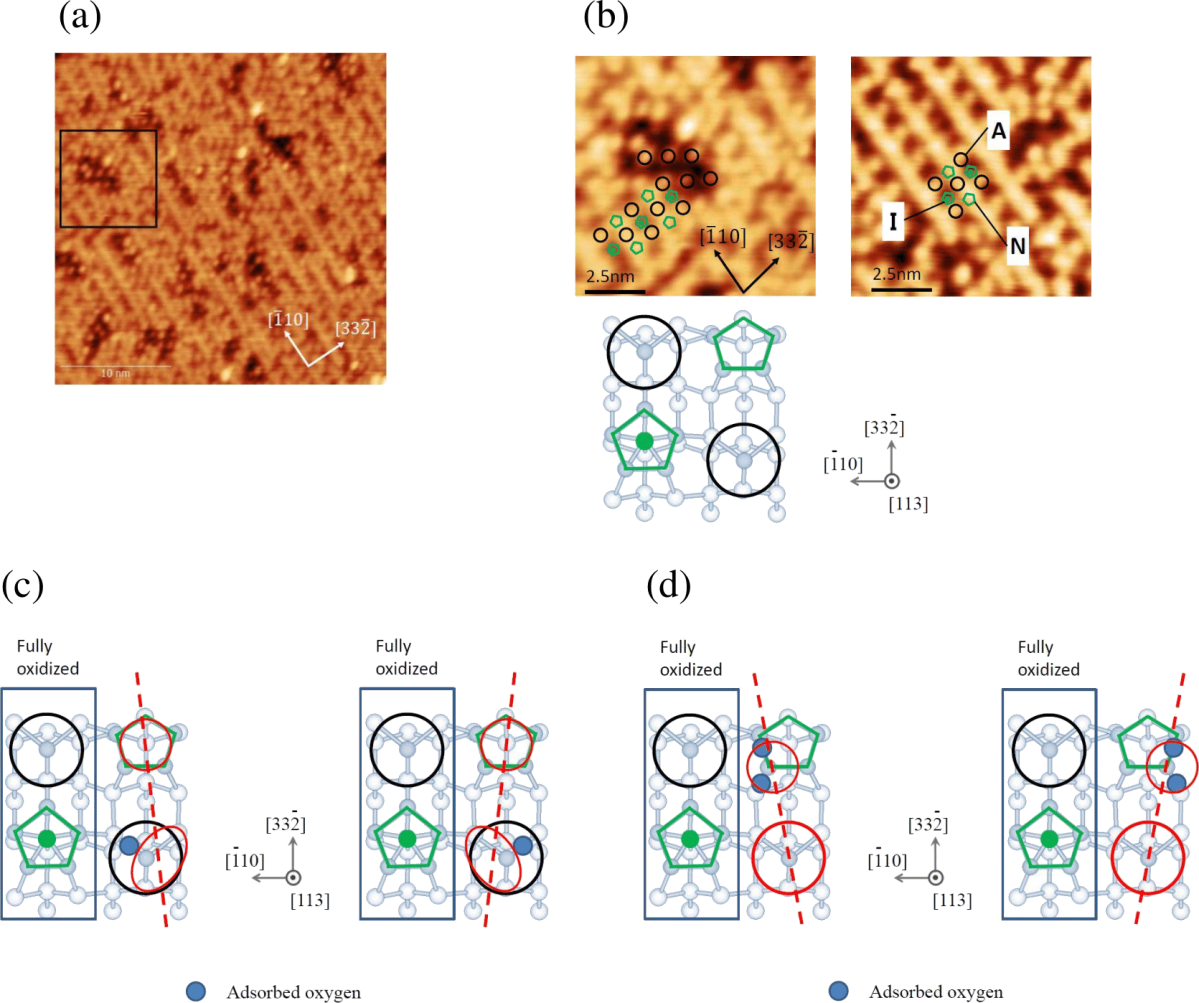
(a) High-resolution STM image of Si(113)-(3 × 2) upon oxidation at an oxygen pressure of 2.6 × 10^−5^ at 820 K, corresponding to the oxidation regime. Total oxygen exposure is 3 L. (b) Magnified image (left) taken at the square in (a) and the corresponding image of a clean Si(113)-(3 × 2) surface at the same scale (right). Here, A represents the adatom site, I represents the i-pentamer site with an interstitial silicon atom, and N represents the n-pentamer site with no interstitial silicon atom. Two types of the possible models (c, d) for the tilted paiers (Pair 1 and Pair 2), as observed in Figure 7b.

The zigzag pattern corresponds to either the pentamer sites or the adatom sites. This means that one of them represents the oxidized position, imaged as a dark depression. To clarify which kind of site the pattern corresponds to, we performed STM observations at a low oxygen exposure of 3 L, as shown in Figure 8a. A magnified image of the square region in Figure 8a is shown in Figure 8b with a reference image of a clean Si(113)-(3 × 2) surface. Based on this observation, we conclude that the zigzag pattern corresponds to the adatom sites. This indicates that the silicon atoms in the pentamer site are preferentially oxidized. It is also possible that the backbond of the adatom is oxidized, while the dangling bond at the adatom site remains unreacted, still imaged as a bright protrusion.

There are three backbonds at each adatom site. The existence of the tilted pairs (Pair 1, Pair 2) may suggest that the adsorption sites of oxygen at the backbond site should have a certain preference. The pair corresponds to one adatom and a pentamer. Here, we assume that one of the three backbonds is oxidized. In such a case, the bright position may shift toward the 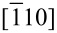 direction or the opposite direction, as shown in Figure 8c. It is also noted that the adjacent adatom and pentamer appear to be fully oxidized, which appear as dark depressions. The existence of a certain oxidation pattern indicates that the preferential adsorption site should be related to the strain induced by the pre-adsorbed oxygen atoms. In the case of Si(111), it has been shown that one oxygen atom at the backbond of a similar adatom site is the common first product for oxidation [20]. Hence, it is reasonable to postulate that a similar structure is formed at the adatom site on Si(113)-(3 × 2). Similarly, it is also reasonable to postulate that the oxidized position inside the pentamer site is antisymmetric to produce a tilted pair. In [18], a dissociative adsorption model of an oxygen molecule at one of the silicon atoms of the n-pentamer site is proposed, as shown in Figure 8d, based on the empty-state image. In this case, the silicon atom adsorbed with two oxygen atoms is shifted upward, and is visualized as a bright protrusion in the STM image. In the present work, we obtained high-resolution images exclusively for the filled state. Here, we simply propose that both are possible structure models to explain the formation of the tilted pairs. We further expect that theoretical investigations would be worthwhile to examine the origin of the formation of such structures.

Finally, we briefly highlight the relation between the findings of this study and technological applications motivated by our previous finding of the remarkably reduced *D*_it_ value on Si(113) [1]. First, we found that silicon atoms were expelled during thermal oxidation, as similar to the case of Si(001) [14]. This clearly indicates that the strain relief mechanism works effectively on Si(113). Second, the atomic model in the early oxidation stage has been elucidated based on high-resolution STM images, as shown in Figure 7. This should encourage further research to establish the structure model towards a thin film regime.

## Conclusion

We investigated three oxidation growth modes – oxidation, etching, and transition modes – in the third of which both oxidation and etching occur. Reaction dynamics in the oxidation of Si(113)-(3 × 2) was observed in real time using VT-STM. Nucleation of the oxide and step-flow etching along the preferential direction have been clearly identified. In the oxidation mode, we observed the unidirectional growth of oxide islands along the 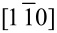 direction. When oxidation and etching occur simultaneously, we deduced that the step-flow etching and faceting due to the effective attractive interaction between the steps take place competitively. We constructed a precise temperature–pressure growth mode diagram for Si(113) as well as for Si(111), and compared the results with those for Si(001) reported in the literature [17]. It is apparent that the oxidation conditions corresponding to the transition region are much more restricted for Si(113) compared with those for Si(001) and Si(111). The transition region, in which both oxidation and etching occur simultaneously, is considered to be related to the enhanced roughness of the oxidized silicon surfaces as reported in the literature [17–18]. Hence, the present results suggest that the relative smoothness of the oxidized surface on Si(113) can be well explained by the limited oxidation conditions for the transition region on this surface. The initial oxygen adsorption pattern could be identified based on a high-resolution STM image. There are several typical patterns for the bright protrusions, which can be explained by the strain-dependent adsorption preference of oxygen.

## Supporting Information

File 1 Animations of the panels of Figures 2, 3, and 5.
